# Development of a high-throughput platform to measure plasmid transfer frequency

**DOI:** 10.3389/fcimb.2023.1269732

**Published:** 2023-10-11

**Authors:** Kepa Arbé-Carton, Ana Rey-Sogo, Nagore Santos-Fernández, Oihane Altube, Carlos Garbisu, Lide Arana, Itziar Alkorta

**Affiliations:** ^1^ Department of Biochemistry and Molecular Biology, University of the Basque Country (UPV/EHU), Bilbao, Spain; ^2^ Department of Conservation of Natural Resources, NEIKER-Basque Institute for Agricultural Research and Development, Basque Research and Technology Alliance (BRTA), Derio, Spain; ^3^ Department of Applied Chemistry, University of the Basque Country (UPV/EHU), Donostia, Spain

**Keywords:** antibiotic resistance, bacterial conjugation, conjugation frequency, conjugation inhibitors, *Escherichia coli*, high-throughput screening platform, horizontal gene transfer

## Abstract

Antibiotic resistance represents one of the greatest threats to global health. The spread of antibiotic resistance genes among bacteria occurs mostly through horizontal gene transfer via conjugation mediated by plasmids. This process implies a direct contact between a donor and a recipient bacterium which acquires the antibiotic resistance genes encoded by the plasmid and, concomitantly, the capacity to transfer the acquired plasmid to a new recipient. Classical assays for the measurement of plasmid transfer frequency (i.e., conjugation frequency) are often characterized by a high variability and, hence, they require many biological and technical replicates to reduce such variability and the accompanying uncertainty. In addition, classical conjugation assays are commonly tedious and time-consuming because they typically involve counting colonies on a large number of plates for the quantification of donors, recipients, and transconjugants (i.e., the bacteria that have received the genetic material by conjugation). Due to the magnitude of the antibiotic resistance problem, it is critical to develop reliable and rapid methods for the quantification of plasmid transfer frequency that allow the simultaneous analysis of many samples. Here, we present the development of a high-throughput, reliable, quick, easy, and cost-effective method to simultaneously accomplish and measure multiple conjugation events in 96-well plates, in which the quantification of donors, recipients, and transconjugants is estimated from the time required to reach a specific threshold value (OD_600_ value) in the bacterial growth curves. Our method successfully discriminates different plasmid transfer frequencies, yielding results that are equivalent to those obtained by a classical conjugation assay.

## 1 Introduction

Antibiotics are indispensable tools for the treatment of bacterial infections. Regrettably, the abuse and inappropriate use of antibiotics have caused the emergence and spread of antibiotic resistant bacteria (ARB) that harbor antibiotic resistance genes (ARGs), as a consequence of antibiotic selective pressure, leading to the loss of efficacy of existing antibiotics (Arana et al., 2021). Some ARB show resistance to several or even all known antibiotics, being currently one of the greatest threats to public health. If this worldwide problem continues to grow as projected, it is estimated that by the year 2050 ARB could kill more than 10 million people (O’Neill, 2016).

ARB can transfer ARGs to other bacteria through horizontal gene transfer (HGT) carried out by three main mechanisms: transformation, transduction, and conjugation. Bacterial conjugation, presumably the most relevant mechanism of HGT, is mediated by mobile genetic elements (MGEs), such as conjugative plasmids and integrative conjugative elements (Jechalke et al., 2014). When mediated by plasmids, the conjugation process involves the transfer of a plasmid from a donor to a recipient bacterium, which then acquires new traits (e.g., antibiotic resistance - AR) as well as the ability to transfer the acquired conjugative element to other bacteria, thereby amplifying the risk of AR spread among bacterial populations. The links between environmental bacteria and human pathogenic bacteria are increasingly being recognized as critical to the AR problem (Jauregi et al., 2021a; Jauregi et al., 2021b; Beltrán de Heredia et al., 2023).

Many studies on the bacterial resistome are nowadays focused on the abundance of ARGs and MGE genes in specific settings and under specific conditions (Jauregi et al., 2021c; Beltrán de Heredia et al., 2023), most likely as a consequence of the development of high-throughput (HT) sequencing techniques which have facilitated the realization of metagenomic studies. While this gene-centric approach is undoubtedly generating important scientific knowledge, we must not forget that genetic information reflects only potentiality and that the real risk of AR dissemination lies in the actual transfer of ARGs to human pathogenic bacteria. Then, conjugation assays for the measurement of plasmid transfer frequency (conjugation frequency) between bacterial cells are essential to assess the risk of AR spread among bacterial populations and, specifically, to investigate the transfer of ARGs from environmental bacteria (e.g., bacteria from agro-livestock settings or wastewater treatment plants) to human bacterial pathogens under different conditions and scenarios. Moreover, plasmid transfer assays can be used to determine the capacity of specific compounds to inhibit bacterial conjugation, in an attempt to define new strategies to control AR spread (Álvarez-Rodríguez et al., 2020a).

Conjugation assays performed to study AR spread have traditionally focused on the transfer frequency of ARG-containing conjugative plasmids. In these experiments, a bacterial population harboring an ARG-containing conjugative plasmid (donor) is put into contact with a recipient bacterial population lacking that specific AR under certain conditions of temperature, salinity, pH, nutrients, presence of contaminants or other compounds (e.g., disinfectants, pharmaceuticals), etc. for a given period of time. Subsequently, the recipient bacteria that have acquired the conjugative plasmid (transconjugants) are selected by growing them in the simultaneous presence of the antibiotic for which the conjugative plasmid confers resistance and the antibiotic for which the recipient bacteria are constitutively resistant. In order to quantify plasmid transfer frequency, the ratio of the number of transconjugants per mL to the number of donors or recipients per mL is calculated.

Classical conjugation experiments are often highly tedious and time-consuming because they typically involve counting colonies (colony-forming units, CFU) on a large number of plates for the quantification of donors, recipients, and transconjugants. Normally, it is necessary to plate a series of dilutions on selective media to achieve an easily countable number of colonies (i.e., to ensure a countable plate) for the quantification of donors, recipients, and transconjugants. Classical conjugation assays are frequently characterized by a high variability and, hence, they require many biological and technical replicates to reduce such variability and the accompanying uncertainty (i.e., to have statistically reliable results) (Lorenzo-Díaz and Espinosa, 2009). All this hampers the performance of studies dealing with the effect of different parameters (e.g., environmental conditions) on plasmid transfer frequency and/or the evaluation of the conjugation capacity of a large number of bacterial strains, since the process is time-consuming, arduous, and cumbersome. It is then critical to develop methods for the rapid quantification of plasmid transfer frequency that allow the simultaneous analysis of many samples to be able to promptly assess the risk of AR spread in a large number of samples or test the capacity of many compounds to inhibit conjugation. Several research groups have developed methodologies for a more rapid quantification of conjugation. Some of these methods are based on the modification of the conjugative plasmid so that its transfer can be evaluated by a fluorescent reporter gene incorporated into the plasmid (Fernández-López et al., 2005; Lorenzo-Díaz and Espinosa, 2009), or by insertion in the conjugative plasmid of genes that provide specific metabolic features (Johnsen and Kroer, 2007). Other authors (Wan et al., 2011) opted for the PCR quantification of a specific gene present in the plasmid which, after conjugation, will also be present in the transconjugants. Kneis et al. (2019) used a methodology for the determination of the presence of transconjugants over time and then compared it to the probability predicted by a mathematical model. These strategies allow a reliable measurement of plasmid transfer frequency but, in general, they lack the set of attributes required for an efficient analysis of the risk of AR dissemination in a large number of samples from complex bacterial communities, that is to say, simplicity, speed, high-throughput, low cost, and reproducibility.

The aim of this study was to develop a HT-conjugation platform to perform plasmid transfer assays in a quick, easy, and cost-effective way. To do so, we combined the 96-filter-well plate method reported by Lorenzo-Díaz and Espinosa (2009) with a procedure that allows the measurement of plasmid transfer frequency from a simple readout (time required to reach a given OD_600_ value in the bacterial growth curve). Plasmid transfer experiments were carried out to search for linear correlations between the number of CFUs of donors, recipients, and transconjugants per mL [log_10_(CFU mL^-1^)] and that OD_600_ readout. The suitability of the HT-conjugation assay was validated through the study of the inhibitory effect of linoleic acid, a compound that affects the transfer of some plasmids but not others.

## 2 Materials and methods

### 2.1 Strains and culture media

Three *Escherichia coli* strains harboring different conjugative plasmids were used as donors in the conjugation assays: *E. coli* DH5α, *E. coli* MS411, and *E. coli* K-12, containing plasmid R388, R1, and pKM101, respectively. Each plasmid belongs to a different incompatibility group (Inc) and confers resistance to the following antibiotic and concentration: R388 to trimethoprim (TMP, 10 µg mL^-1^), R1 to kanamycin (KAN, 40 µg mL^-1^), and pKM101 to ampicillin (AMP, 50 µg mL^-1^) (**Table 1**). These three strains and plasmids have extensively been used in conjugation studies. Plasmid R1, an F-like conjugative plasmid of the IncFII incompatibility group, has long served as a model system for studying conjugative plasmid biology (Cox and Schildbach, 2017). Plasmid R1, as well as plasmids R388 (a broad-host-range IncW plasmid that contains one of the simplest conjugation systems at the genetic level) and pKM101 (an IncN plasmid), were selected by Fernández-López et al. (2005) for their study on the inhibition of conjugation by unsaturated fatty acids, finding out that conjugation mediated by the first two was inhibited by linoleic acid, unlike that mediated by plasmid pKM101 (see below experiment on inhibition by linoleic acid). *Escherichia coli* plasmids R388 and pKM101, whose conjugation systems are well-characterized (Christie, 2016), are phylogenetically closely related, but more distantly related to *E. coli* F plasmid (González-Rivera et al., 2019). *Escherichia coli* HMS174, a strain with intrinsic chromosomal resistance to rifampicin (RIF, 50 µg mL^-1^), was used as the only recipient in the conjugation experiments (**Table 1**).

**Table 1:** *Escherichia coli* strains and plasmids used in the conjugation experiments.

	*E. coli* strain	Conjugative plasmid	Size (kb)	Inc Group	Antibiotic resistance^#^ (μg mL^-1^)	Reference
**Donors**	DH5α	R388	33.9	IncW	10 TMP	Álvarez-Rodríguez et al., 2020b
	MS411	R1	97.6	IncFII	40 KAN	Cox and Schildbach, 2017
	K-12	pKM101	35.4	IncN	50 AMP	Langer et al., 1981
**Recipient**	HMS174	*			50 RIF	Campbell et al., 1978

*Not applicable.

^#^For donor strains, antibiotic resistance was conferred by their respective conjugative plasmid. The recipient strain contains intrinsic chromosomal resistance to rifampicin.

Lysogeny Broth (LB) culture medium (Bertani, 1951), supplemented with the corresponding antibiotic, was used for *E. coli* growth. For the donor bacteria, LB medium was supplemented with 10 µg TMP mL^-1^ (Merck, Darmstadt, Germany), 40 µg KAN mL^-1^ (PanReac AppliChem, Darmstadt, Germany), and 50 µg AMP mL^-1^ (PanReac AppliChem) for *E. coli* DH5α, *E. coli* MS411, and *E. coli* K-12, respectively. For the recipient bacteria, LB medium was supplemented with 50 µg RIF mL^-1^ (PanReac AppliChem) (**Table 1**). In both the classical and the HT-conjugation method, quality control tests for donors, recipient, and transconjugants were included, based on the expected antibiotic resistances, to exclude the possibility of bacterial contaminants interfering with the results.

### 2.2 Classical conjugation assay

**Figure 1:**
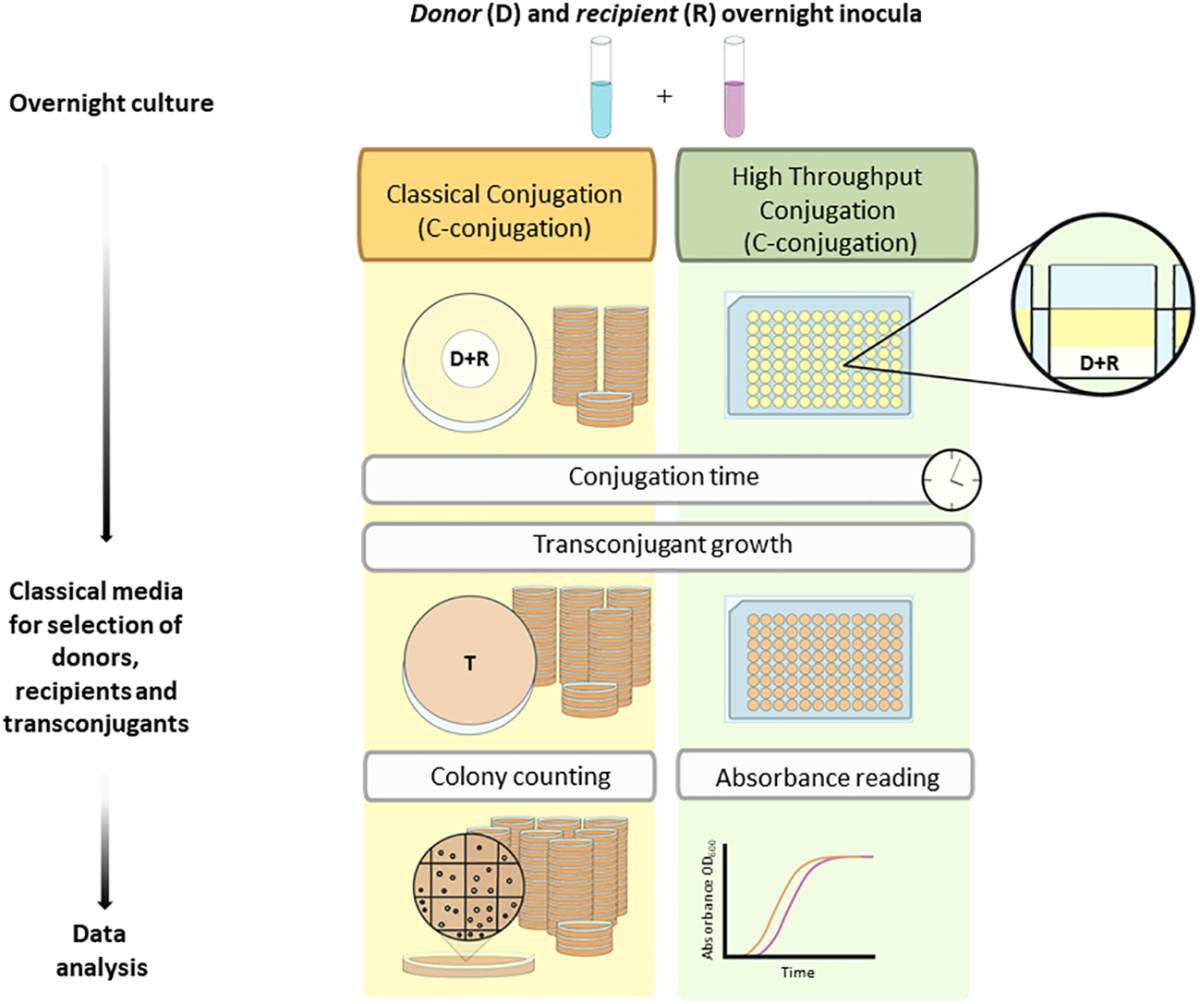
Scheme of the plasmid transfer frequency assays. Classical conjugation assay: one single conjugation event can be carried out in each Petri dish. For quantification of plasmid transfer frequencies by colony counting, serial dilutions must be plated in separate LB-agar plates supplemented with the antibiotic used for selection. High-throughput conjugation assay: 96 conjugation events can be carried out simultaneously in a 96-filter-well plate. Quantification of plasmid transfer frequencies can be inferred by the linear correlation between log_10_(CFU mL^-1^) values of donors, recipients, and transconjugants and a simple readout, i.e., the time needed to reach a specific OD_600_ value (time to threshold).

In order to measure plasmid transfer frequencies, classical bacterial conjugation assays (C-conjugation) (**Figure 1**) were performed following Álvarez-Rodríguez et al. (2020b) with some modifications. Briefly, donor and recipient strains were grown overnight in 3.5 mL of LB supplemented with the corresponding antibiotic at 37 °C and orbital shaking at 220 rpm (**Table 1**). Cultures were then diluted with LB to reach an optical density (OD) value of 0.6 at 600 nm (OD_600_ = 0.6). Next, 100 μL of the donor culture was mixed with 100 μL of the recipient culture. The mixture was centrifuged at 6,000 *x g* for 2 min, and the pellet was resuspended in 50 µL of LB without antibiotic. This 50 µL suspension was deposited onto a Millipore filter (0.22 μm pore size) previously placed on a pre-warmed LB-agar plate without antibiotic. After 6 h at 37 °C, cells were detached from the filter by adding 3 mL of 0.8% (w/v) NaCl and vortexing for 30 s. Subsequently, 50 µL of this bacterial suspension was plated on LB-agar with the corresponding selective antibiotic(s) for recipients and transconjugants. Plates were incubated at 37 °C for 24 or 48 h depending on the specific growth rate of each strain. These differences in growth rate are due to the different fitness costs induced by the different conjugative plasmids, with a concomitant effect on bacterial growth rates (Prensky et al., 2021) (**Table 2**). Plasmid transfer frequency was quantified as the ratio of the number of CFU of transconjugants per mL to the number of CFU of recipients per mL (CFU_T_ mL^-1^/CFU_R_ mL^-1^). All assays were performed with five biological replicates and three technical replicates.

**Table 2:** Experimental conditions for donors, recipient, and transconjugants.

	Strain	Antibiotic for selection (μg mL^-1^)	Growth time^#^ (h)
**Donors**	*E. coli* DH5α (R388)	10 TMP	48
	*E. coli* MS411 (R1)	40 KAN	24
	*E. coli* K-12 (pKM101)	50 AMP	24
**Recipient**	*E. coli* HMS174	50 RIF	24
Transconjugants*	T_R388_	10 TMP+50 RIF	48
	T_R1_	40 KAN+50 RIF	24
	T_pKM101_	50 AMP+50 RIF	24

*T_R388_, transconjugant that acquired plasmid R388; T_R1_, transconjugant that acquired plasmid R1; T_pKM101_, transconjugant that acquired plasmid pKM101.

^#^Bacteria were grown for 24 or 48 hours depending on their growth rate.

### 2.3 High-throughput conjugation assay

For the high-throughput bacterial conjugation assays (HT-conjugation) (**Figure 1**), donor and recipient bacteria were grown overnight in 3.5 mL of LB supplemented with the corresponding antibiotic at 37 °C and orbital shaking at 220 rpm (**Table 1**). Cultures were then diluted with LB to an OD_600_ = 0.6. Next, 100 μL of the donor culture was mixed with 100 μL of the recipient culture. The mixture was centrifuged at 6,000 *x g* for 2 min, and the pellet was resuspended in 50 µL of LB without antibiotic. This 50 µL suspension was deposited in a well of a sterile 96-filter-well plate (0.22 µm) (Merck, Darmstadt). The plate was adapted to a vacuum manifold (MultiScreenHTS) and vacuum (between 5-10 mm Hg) was applied to attach the cells to the filter, following Lorenzo-Díaz and Espinosa (2009). Then, 150 μL of LB without antibiotic was added to the well and the plate was incubated at 37°C for 6 h onto a LB-agar rectangular plate. For those experiments aimed at determining the effect of linoleic acid on plasmid transfer frequencies, the corresponding amounts of linoleic acid were added to the wells to reach the concentrations to be tested. After the 6-hour conjugation time, 150 μL of each well was removed by vacuum filtration as described above. For cell detachment from the filter, 200 μL of 0.8% (w/v) NaCl was added to each well and bacteria were resuspended by pipette mixing 10 times. Next, 10 μL of this suspension was inoculated into a sterile 96-well flat-bottomed microplate (TC-Plate 96 Well, Standard, R, Sarstedt, Nümbrecht, Germany), and 140 μL of LB supplemented with the corresponding antibiotic for the selection of recipients and transconjugants was added (**Table 2**). Plates were incubated at 37°C for 24 h in an Infinite MNano plate reader (Tecan, Männedorf, Switzerland) and OD_600_ readings were taken every 10 min to obtain the growth curves for recipients and transconjugants. All assays were performed with five biological replicates and three technical replicates. Plasmid transfer frequency was also quantified as the ratio of the number of CFU of transconjugants per mL to the number of CFU of recipients per mL (CFU_T_ mL^-1^/CFU_R_ mL^-1^). In this case, instead of counting colonies on LB-agar plates, CFU_T_ mL^-1^ and CFU_R_ mL^-1^ values were estimated from the abovementioned growth curves.

**Figure 2:**
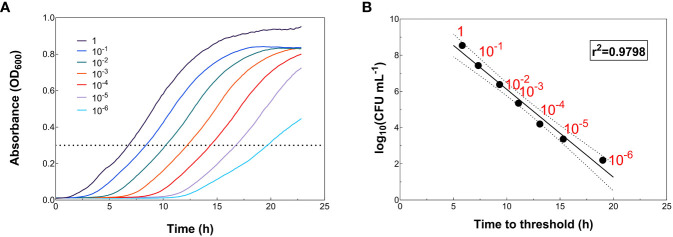
Correlation between log_10_(CFU mL^-1^) values and the time needed to reach an OD_600_ = 0.3 (time to threshold) for recipient *E. coli* HMS174 strain. **(A)** Growth curves of the different bacterial dilutions. The dotted line marks the threshold value. **(B)** Linear regression between log_10_(CFU mL^-1^) values obtained by colony counting in LB-agar plates supplemented with 50 μg RIF mL^-1^ and the time at which each growth curve reaches an OD_600_ = 0.3 (time to threshold). The solid line represents the best data fit. Dashed lines correspond to the margin of error of the data with a 95% confidence interval.

**Figure 3:**
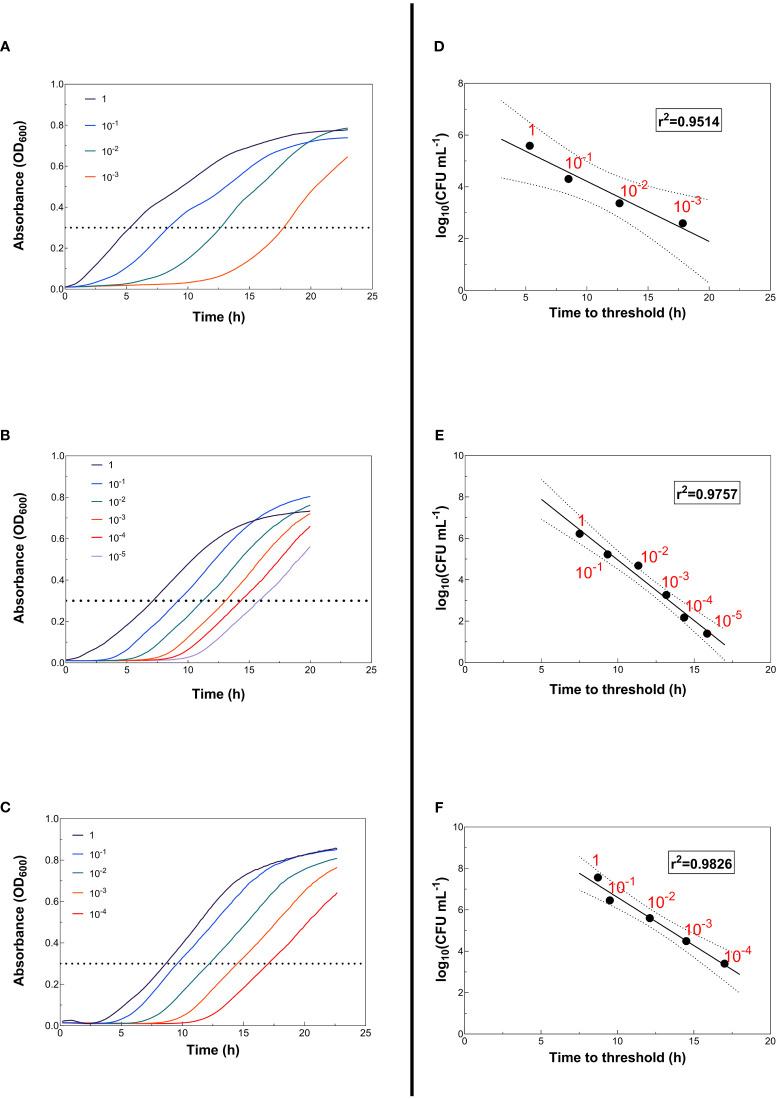
Correlation between log_10_ (CFU mL^-1^) values and the time needed to reach an OD_600_ = 0.3 (time to threshold) for transconjugants. **(A)** T_R388_; **(B)** T_R1_; **(C)** T_pKM101_) Growth curves of the different bacterial dilutions. The dotted line marks the threshold value. **(D)** T_R388_; **(E)** T_R1_; **(F)** T_pKM101_) Linear regression between log_10_ (CFU mL^-1^) values obtained by colony counting in LB-agar plates supplemented with the appropriate antibiotic for transconjugant selection and the time at which each growth curve reaches an OD_600_ = 0.3 (time to threshold). The solid line represents the best data fit. Dashed lines correspond to the margin of error of the data with a 95% confidence interval.

Linear correlations between the time needed to reach a given OD_600_ value in the growth curves of recipients and transconjugants and the number of CFU mL^-1^ of recipients and transconjugants (on a logarithmic scale) (**Figures 2B**; **3D–F**) were searched for. To obtain the growth curves, serial dilutions of the recipient and transconjugant suspensions described above were prepared with LB medium (**Figures 2A**; **3A–C**). Depending on the growth rate of each strain, dilutions ranged from 10^-1^ to 10^-5^ for recipient and from 10^-1^ to 10^-6^ for transconjugant suspensions. Dilutions for plasmid R388-containing transconjugants (T_R388_) ranged from 10^-1^ to 10^-3^; dilutions for plasmid R1-containing transconjugants (T_R1_) ranged from 10^-1^ to 10^-5^; and dilutions for plasmid pKM101-containing transconjugants (T_pKM101_) ranged from 10^-1^ to 10^-4^. Growth curves were obtained in an Infinite MNano plate reader as described above. Dilutions of recipients and transconjugants were plated on LB-agar medium supplemented with the corresponding selective antibiotics (**Table 2**) to determine the number of CFU mL^-1^. When required to ensure a countable plate, suspensions were further diluted to the appropriate concentration at which CFU mL^-1^ could be easily counted in the LB-agar plates.

Calibration curves that correlate the time required to reach a predetermined threshold value (“time to threshold”) with inoculum size can be constructed, so that unknown initial cell densities can be predicted from time to threshold values (Bethke et al., 2020). In our study, it was observed that, at an OD_600_ = 0.3, all cultures had reached the exponential phase (**Figures 2A**; **3A–C**). Then, the CFU_T_ mL^-1^ and CFU_R_ mL^-1^ values obtained in the LB-agar plates inoculated with the abovementioned dilutions were represented against the time (in hours) needed to reach that threshold value (OD_600_ = 0.3) in the growth curves, i.e., “time to threshold” (**Figures 2**; **3D–F**). The resulting plots can be used as calibration plots to calculate the number of recipients and transconjugants of unknown samples according to the time required to reach the threshold value.

The suitability of the HT-conjugation assay was validated through the study of the inhibitory effect of linoleic acid, a compound that has been reported to affect the transfer of some conjugative plasmids but not others (Fernández-López et al., 2005). Using the HT-conjugation assay, plasmid transfer frequencies were measured in the presence of different concentrations of linoleic acid (0, 1, 10, 50, 100, 200, 300, and 500 µM). Not all of these concentrations were tested for all cases (concentrations of linoleic acid were adjusted depending on the observed degree of inhibition for each case). According to preliminary studies (data not shown), none of these linoleic acid concentrations affected the growth of either the donor or recipient cells.

### 2.4 Graphical representations and statistical analysis

Bacterial growth curves (**Figures 2A**; **3A–C**) and calibration plots (**Figures 2B**; **3D–F**) were graphed using GraphPad Prism 9 (GraphPad Software, San Diego, CA). Statistical analyses, carried out to search for statistically significant differences in plasmid transfer frequencies between C-conjugation and HT-conjugation assays, were performed using the two-tailed paired Student’s t-test using GraphPad Prism 9 (GraphPad Software), where p < 0.05 was considered to be significant.

## 3 Results

### 3.1 Development of the high-throughput platform

In conjugation assays, plasmid transfer frequency is often quantified as the ratio of the number of CFU of transconjugants per mL to the number of CFU of recipients (or donors) per mL, which usually requires counting colonies on a large number of plates. Here, instead of counting colonies on selective medium plates, CFU_T_ mL^-1^ and CFU_R_ mL^-1^ values were estimated from the growth curves of recipient and transconjugant bacteria. Such estimations were based on linear correlations between the number of CFU mL^-1^ (on a logarithmic scale) and the time needed to reach a specific OD_600_ value in the growth curves (**Figures 2**, **3**). Initially, serial dilutions of recipient and transconjugant suspensions were prepared and plated on LB-agar medium supplemented with the corresponding selective antibiotics to determine the number of CFU mL^-1^ of recipient and transconjugants (**Table 2**). These recipient and transconjugant suspensions were used to obtain the growth curves (OD_600_). As shown in **Figures 2** (2A for recipient) and **3** (3A–C for transconjugants), at an OD_600_ = 0.3, all cultures had reached the exponential phase. Then, the time required to reach an OD_600_ = 0.3 was set as threshold value. As shown in **Figures 2** (2B for recipient) and **3** (3D–F for transconjugants), very good inverse linear correlations between log_10_(CFU mL^-1^) values and time to threshold values were detected: r^2^ values ranged from 0.95 to 0.99. Maximum OD_600_ values reached by the different bacterial cultures were around 0.75-0.80 and the duration of the lag phase ranged from close to zero for larger inoculum sizes to 10-12 h for smaller inoculum sizes. The good regression parameters obtained for all cases indicate that the calibration plots can be used to calculate the number of recipients (**Figure 2B**) and transconjugants (**Figures 3D–F**) from “time to threshold” values.

**Figure 4:**
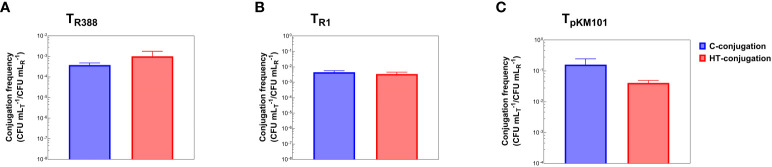
Plasmid transfer frequencies of plasmids R388 **(A)**, R1 **(B)**, and pKM101 **(C)** measured by the classical conjugation (C-conjugation) versus the high-throughput conjugation (HT-conjugation) assay. In the C-conjugation assay, plasmid transfer frequencies were obtained by colony counting (CFU mL^-1^) in LB-agar plates. In the HT-conjugation assay, plasmid transfer frequencies were obtained according to the calibration plots used to calculate the number of recipients and transconjugants (CFU mL^-1^) inferred from the time to threshold value. Plasmid transfer frequency was defined as the ratio between the number of CFU mL^-1^ of transconjugants and the number of CFU mL^-1^ of recipients (CFU_T_ mL^-1^/CFU_R_ mL^-1^). Results are the mean ± SE of five independent experiments performed in triplicate. Differences between the two methods were not statistically significant (p > 0.05).

The HT-conjugation assay was validated by comparison with the results obtained with the classical conjugation assay (C-conjugation). To this purpose, once the HT-conjugation assay had been designed and the calibration plots [based on linear regressions between log_10_(CFU mL^-1^) values and time to threshold values] for recipients and transconjugants had been established, it was evaluated whether the plasmid transfer frequencies obtained with the HT-conjugation assay were comparable to those obtained with the C-conjugation assay. To this goal, transfer frequencies of plasmids R388, R1, and pKM101 were measured by both assays, finding out similar values for the three plasmids. Conjugation frequency values for plasmid R388 obtained with the C-conjugation and HT-conjugation assays were 2.27 x 10^-4^ ± 4.12 x 10^-5^ and 4.69 x 10^-4^ ± 5.22 x 10^-5^, respectively. For plasmid R1 conjugation frequency, the values obtained by both assays were 4.49 x 10^-3^ ± 5.95 x 10^-4^ (C-conjugation) and 1.59 x 10^-3^ ± 2.15 x 10^-4^ (HT-conjugation), and for plasmid pKM101 were 2.03 x 10^-1^ ± 3.05 x 10^-2^ (C-conjugation) and 1.04 x 10^-1^ ± 3.35 x 10^-2^ (HT-conjugation) (**Figure 4**). According to the two-tailed paired Student’s t-test, the observed differences between the values obtained from both assays for the three plasmids were not statistically significant (differences were in the range of the variability observed within each assay). In other words, the observed differences in transfer frequencies of the three plasmids between C-conjugation and HT-conjugation assays were not biologically relevant, pointing out to the suitability of the HT-conjugation assay presented here.

### 3.2 Validation of the high-throughput platform with linoleic acid as inhibitor

**Figure 5:**
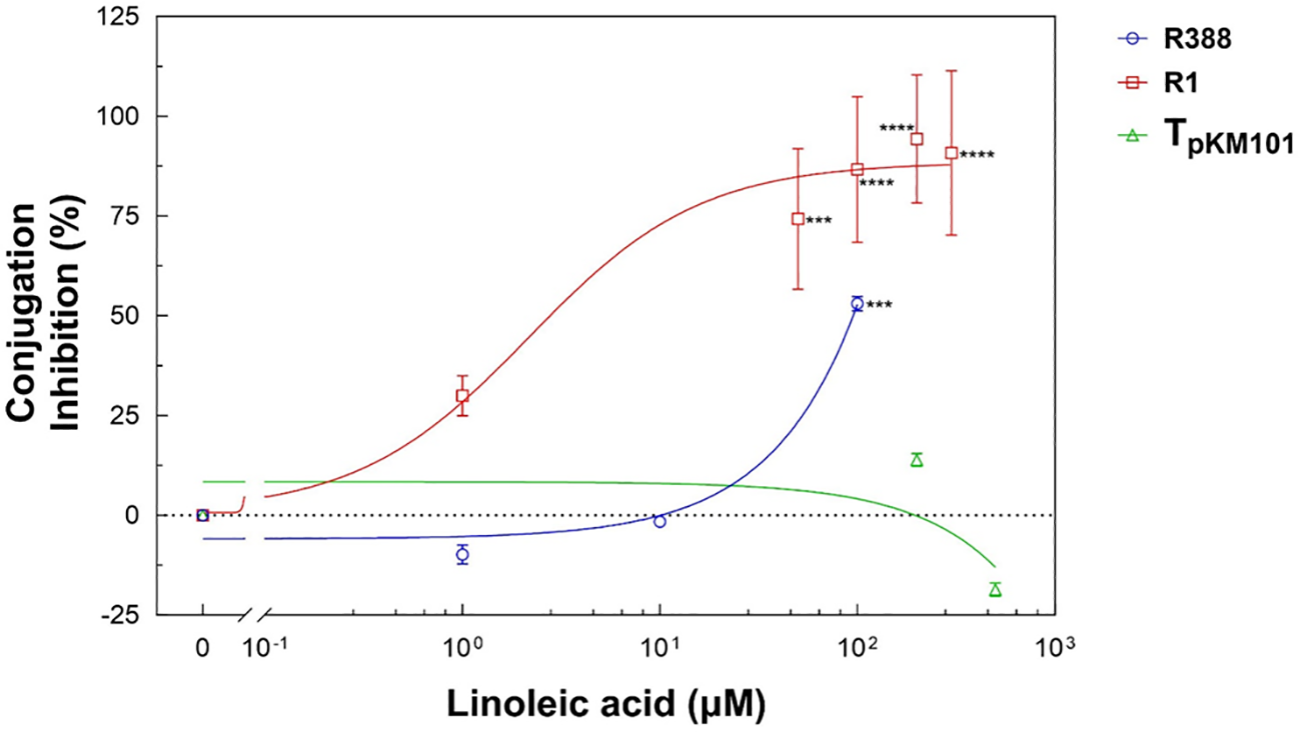
Effect of linoleic acid on plasmid transfer frequency measured by the high-throughput conjugation assay. Data are expressed as percentage of inhibition relative to control value (0% = no linoleic acid). Results are the mean ± SE of five independent experiments performed in triplicate. The statistical significance of the observed differences with respect to the control (no linoleic acid) was analyzed by Student’s t test (***p <0.005, ****p < 0.001).

Once it had been verified that the HT-conjugation assay could quantify the transfer frequency of the three plasmids, its capacity to differentiate expected differences in conjugation frequencies, according to previously reported knowledge, was tested. To do so, a compound that has been reported to inhibit the transfer of some conjugative plasmids but not others, i.e., linoleic acid (Fernández-López et al., 2005), was selected. Linoleic acid does inhibit conjugation mediated by plasmid R388 and F plasmids (such as plasmid R1), but not by plasmids pKM101, R6K, and RP4 (Fernández-López et al., 2005). R388, R1, and pKM101 plasmid transfer frequencies were measured in the presence of different concentrations of linoleic acid using the HT-conjugation assay, finding out that it did detect the inhibitory effect of linoleic acid on the conjugation frequency of plasmids R388 and R1 (**Figure 5**). Furthermore, the transfer frequency of plasmid pKM101 was not inhibited by linoleic acid (**Figure 5**). The values of plasmid transfer frequencies for each plasmid in the presence of different concentrations of linoleic acid are shown in **Table 3**. The transfer frequency of plasmid R388 decreased from 5.84 x 10^-6^ ± 2.94 x 10^-7^ in the absence of linoleic acid to 2.72 x 10^-6^ ± 4.44 x 10^-8^ in the presence of 100 µM linoleic acid; thus, linoleic acid inhibited the transfer of plasmid R388 by approximately 50% (statistical significance: ***p < 0.005). For plasmid R1, the frequency of conjugation decreased from 1.81 x 10^-3^ ± 9.07 x 10^-5^ in the absence of linoleic acid to 4.66 x 10^-4^ ± 4.94 x 10^-5^, 2.41 x 10^-4^ ± 2.28 x 10^-5^, 1.03 x 10^-4^ ± 7.86 x 10^-6^, and 1.67 x 10^-4^ ± 1.07 x 10^-5^ in the presence of 50, 100, 200, and 300 µM linoleic acid, respectively. All the differences were statistically significant. Then, an inhibition of almost 75% was observed at 50 µM linoleic acid and above. Finally, plasmid pKM101 conjugation frequencies measured with the HT-conjugation assay showed no statistically significant differences in the presence versus absence of linoleic acid, reflecting the fact that this compound does not inhibit the transfer of plasmid pKM101, in agreement with Fernández-López et al. (2005). It was concluded that the HT-conjugation assay can differentiate expected differences (according to previously reported knowledge) in conjugation frequencies.

**Table 3:** Effect of different concentrations of linoleic acid on conjugation frequencies of plasmids R388, R1, and pKM101.

	Linoleic acid (µM)	Conjugation frequency (CFU_T_ mL^-1^/CFU_R_ mL^-1^)
**R388**	0	5.84 x 10^-6^ ± 2.94 x 10^-7^
	1	6.42 x 10^-6^ ± 6.99 x 10^-7^
	10	5.94 x 10^-6^ ± 1.79 x 10^-7^
	100	2.72 x 10^-6^ ± 4.44 x 10^-8^
**R1**	0	1.81 x 10^-3^ ± 9.07 x 10^-5^
	1	1.27 x 10^-3^ ± 9.49 x 10^-5^
	50	4.66 x 10^-4^ ± 4.94 x 10^-5^
	100	2.41 x 10^-4^ ± 2.28 x 10^-5^
	200	1.03 x 10^-4^ ± 7.86 x 10^-6^
	300	1.67 x 10^-4^ ± 1.07 x 10^-5^
**pKM101**	0	1.42 x 10^-2^ ± 8.01 x 10^-4^
	200	1.22 x 10^-2^ ± 6.36 x 10^-4^
	500	1.68 x 10^-2^ ± 6.81 x 10^-4^

Mean values ± SE of five independent experiments performed in triplicate.

## 4 Discussion

Antibiotic resistance phenotypes rely on the presence of ARGs in bacterial chromosomes and/or extrachromosomal genetic elements. ARG-harboring bacteria can transfer ARGs to other bacteria by HGT mediated by MGEs. Then, we must closely monitor the possibility of ARG transfer from non-pathogenic environmental bacteria to human pathogenic bacteria (Perry and Wright, 2013; von Wintersdorff et al., 2016). Conjugation, the main mechanism responsible for AR dissemination (Frost et al., 2005), requires a physical contact between the donor cells carrying the plasmid and the recipient cells to then generate the transconjugants (Ochman et al., 2000), which can later contribute to plasmid transfer to new recipients. When conjugative plasmids enter a bacterium, they often trigger its SOS response, driving the swapping of integron cassettes and stimulating the generation of multiresistant strains (Baharoglu et al., 2010). Moreover, antibiotic treatment has been suggested to promote HGT, but this fact is still a matter of much debate (Møller et al., 2017).

Due to its crucial role in AR spread, conjugation has been extensively studied from different points of view and using a variety of experimental approaches (Huisman et al., 2022). The quantification of plasmid transfer frequency has been widely applied to assess the risk of AR spread (González-Plaza et al., 2019; Macedo et al., 2022). Nonetheless, there is no generally accepted method to quantify conjugation frequency (Huisman et al., 2022). Being aware of this limitation, Huisman et al. (2022) compared a variety of methods, finding out that conjugation frequency estimates strongly depend on time of measurement, initial population densities, and the ratio of donor to recipient populations (differences in growth rates also affected conjugation frequency estimates). The quantification of plasmid transfer has mostly focused on a few laboratory strains, which might be poor models for natural bacterial populations (Hobman et al., 2007; Dimitriu et al., 2019). For instance, *E. coli* K-12 has been questioned as a model organism since the current versions of this strain appear to be quite distinct from any original, wild-type, free-living ancestor and, besides, it is usually studied under conditions far removed from its natural habitats (Hobman et al., 2007).

In order to carry out experimental work on a large number of environmental and/or clinical samples, it is crucial to have a method that can accurately, quickly, and cost-effectively quantify plasmid transfer frequency. Classical laboratory assays for the determination of conjugation frequency are commonly highly tedious and time-consuming. Besides, they are poorly reproducible and, hence, it is often necessary to increase the number of replicates for statistical robustness. In consequence, the high number of biological and technical replicates that are required to provide statistical robustness substantially complicates the development of experiments involving a large number of samples and/or experimental conditions (Kneis et al., 2019). In the case of environmental samples, methodological strategies based on the modification of the conjugative plasmid to assess its transfer (Fernández-López et al., 2005; Johnsen and Kroer, 2007; Lorenzo-Díaz and Espinosa, 2009) are often not feasible, as the very nature of the samples and their diversity make these approaches a daunting task. Within this context, Alalam et al. (2020) designed a high-throughput platform for the measurement of AR plasmid conjugation and the identification of all the *E. coli* genes that control F-plasmid donation, as well as novel conjugation genes not previously linked to AR transmission.

Inspired by the work of Lorenzo-Díaz and Espinosa (2009), conjugation assays were carried out here in 96-filter-well plates. Filter mating provides high cell density and close proximity for donor and recipient cells, facilitating the formation of mating pairs for plasmid transfer (Lim et al., 2008; Aviv et al., 2016). After conjugation, instead of quantifying the number of recipients and transconjugants by plating a series of dilutions on LB-agar plates supplemented with the corresponding selective antibiotics, sterile 96-well flat-bottomed microplates were used to record the growth curves of the diluted cell suspensions in the appropriate selective media. The quantification of recipients and transconjugants was carried out by defining a “time required to reach a predetermined threshold value of an easily measurable parameter” (OD_600_), following Bethke et al. (2020). From the growth curves, it was observed that at an OD_600_ = 0.3 all cultures had reached the exponential phase, and the time taken for each culture to reach this OD_600_ value was established as “time to threshold”. A linear negative relationship between “time to threshold” and inoculum size of recipients and transconjugants, measured as number of CFU per mL of inoculum in LB-agar plates [expressed as log_10_(CFU mL^-1^)], was found. This negative linear correlation between the log of the initial inoculum size [expressed as log_10_(CFU mL^-1^)] and the “time to threshold” value, observed for the range of serial dilutions used here, is mostly, if not entirely, due to the decrease in the initial number of cells growing exponentially (i.e., the increase in the “time to threshold” value observed in the diluted cultures is a direct consequence of the lower initial concentration of cells). The negative linear correlation observed in exponentially growing cells between the log of the initial inoculum size [log_10_(CFU mL^-1^)] and the “time to threshold” value was then used to determine conjugation frequencies. The HT conjugation procedure presented here is valid as long as the experimental values of the “time to threshold” are within the linear range.

For donors, a linear relationship was also observed (data not shown) but, since the quantification of plasmid transfer frequencies was referred here to a common recipient strain (*E. coli* HMS174), only the calibration plots for recipients and transconjugants are shown. The linear regression parameters of all the calibration plots were good, with r^2^ values ranging from 0.95 to 0.99. All the experimental points of the calibration plots were within a margin of error below the 95% confidence interval. The calibration plots were used to infer the number of recipients and transconjugants from the “time to threshold” values obtained from the growth curves, thus allowing the simultaneous assessment of 96 conjugation events.

Donor and recipient cells were put into contact for 6 h (conjugation time = 6 h). When comparing data among conjugation studies, the duration of the conjugation time must be taken into consideration, as conjugation frequency curves often resemble bacterial growth curves, with a lag phase occurring after the initial mixing of donor and recipient cells, followed by a period of increasing conjugation that normally ends in a plateau (Andrup and Andersen, 1999; Fox et al., 2008; Wan et al., 2011; Headd and Bradford, 2020). Although most studies on conjugation frequency are focused on cultures of bacteria growing for short periods of time, under natural conditions bacteria often grow episodically and at much lower rates than those observed in the laboratory. Headd and Bradford (2020) studied the dynamics of conjugation of *E. coli* cells harboring plasmid pUUH239.2 (an IncFII plasmid containing multiantibiotic- and metal-resistance genes) over a 24-h period in the presence and absence of antibiotics, finding out that (i) conjugation occurred only in a narrow time frame when *E. coli* cells were transitioning from a growing to a non-growing phase; and (ii) the conjugation plateau developed because of a lack of capable donor cells. Their results suggest that episodic growth in nutrient-depleted environments (as it is frequently the case in many environmental settings) could result in more conjugation than sustained growth in a nutrient rich environment (Headd and Bradford, 2020). Conjugation dynamics are basically governed by two kinetic processes: the rate of gene transfer (or conjugation efficiency) and the relative growth rate of transconjugants (or the growth dynamics) (Prensky et al., 2021). The hours immediately following conjugation can play a critical role in both short- and long-term plasmid prevalence; actually, newly generated transconjugants can exhibit transient reduced growth rates and/or prolonged lag times, pointing out to the presence of an increased burden immediately after conjugation (plasmid acquisition cost) which occurs independently of long-term fitness effects (Prensky et al., 2021). It has been reported (Newbury et al., 2022) that the degree to which a plasmid spreads through a host community (both the number of host species in which it is found and the evenness of its prevalence between host species) can be explained as a direct result of the effect the plasmid has on its hosts’ population growth rate. In any event, many more studies on the dynamics of bacterial conjugation under conditions that mimic real settings are needed to properly assess the risk of AR dissemination.

Huisman et al. (2022) emphasized that conjugation methods, based on the ratio between population densities, e.g., transconjugants/recipients or transconjugants/donors, vary as a function of initial population densities, initial donor to recipient ratios, and the length of the conjugation assay (Simonsen et al., 1990; Zhong et al., 2012). In a study on plasmid transfer among *E. coli* isolates harboring one to five plasmids, Darphorn et al. (2022) found that, under standardized conditions, plasmid transfer rates varied by a factor of almost 10^6^, depending on the specific recipient/donor strain combination. In consequence, to allow for comparisons among studies, comprehensive information on the experimental conditions and strain growth rates must always be included (Huisman et al., 2022). On the other hand, conjugation efficiency is influenced by the taxonomic relatedness between donor and recipient bacteria (Hunter et al., 2008). Dimitriu et al. (2019) studied the importance of genetic similarity between naturally co-occurring *E. coli* isolates for plasmid transfer, finding out that plasmid transfer was strongly biased toward clone-mates, but not correlated to genetic distance when donors and recipients were not clone-mates. Alderliesten et al. (2020) carried out a meta-analysis on conjugation frequencies from *E. coli* donors to various recipient species and observed that taxonomic relatedness was limiting conjugation in liquid matings, but not in filter matings, suggesting that taxonomic relatedness might not be a limiting factor for conjugation in those environments where bacteria are fixed in space.

Although a wide variety of biotic and abiotic factors can affect plasmid transfer (e.g., temperature, nutrient concentration, pH, moisture, cell physiology, population densities, type of plasmid, type of donor and recipient, growth on surfaces versus growth in well-mixed liquids), the transfer of conjugative plasmids in biofilms is of paramount importance (Águila-Arcos et al., 2017), as most bacteria live in biofilms, microcolonies or similar forms of clumped cells with an explicit spatial structure (Stalder and Top, 2016). Biofilm growth can promote the initial transfer of some plasmids, but also limit further plasmid invasion into the population or community (Stalder and Top, 2016). A much less explored factor that can also affect plasmid transfer is the ecological growth strategy (K-strategists, r-strategists, oligotrophs, copiotrophs) of donors and recipients (Varner et al., 2022). In any case, the monitoring of environmental HGT remains challenging mainly due to cultivation bias (Pallares-Vega et al., 2021).

The high-throughput platform presented here has been validated according to two different approaches. First, the transfer frequencies of three plasmids belonging to different incompatibility groups (R388, R1, pKM101) were quantified by both the classical (C-conjugation) and the high-throughput (HT-conjugation) assay. In all cases, similar values were obtained by the C-conjugation versus the HT-conjugation assay. Due to the variability of the conjugation process itself, the observed differences between both methods were smaller than those found in different experiments carried out with the same method. Secondly, it was tested whether the HT-conjugation assay could differentiate expected differences in conjugation frequencies. For this purpose, the effect of linoleic acid, a compound that inhibits the transfer of some conjugative plasmids but not others (Fernández-López et al., 2005), was determined. When HT-conjugation experiments were performed with plasmids R388 and R1, for which the inhibition of conjugation by linoleic acid has been reported (Fernández-López et al., 2005), the expected inhibition was observed. When the frequency of conjugation was measured with plasmid pMK101, linoleic acid showed no effect on such frequency, as previously reported (Fernández-López et al., 2005).

From all of the above, it was concluded that the high-throughput conjugation platform developed here is a suitable, reliable, time and money saving, methodological tool for the simultaneous assessment of plasmid transfer frequency in a wide number of environmental and/or clinical samples. This high-throughput platform can, for instance, be used to simultaneously screen a large number of molecules or compounds as potential inhibitors of bacterial conjugation, in an attempt to minimize the risk of AR dissemination. Despite these promising results, it must be taken into consideration that this study has been carried out using only three *E. coli* strains (each harboring a different plasmid) as donor cells and one *E. coli* strain as recipient. For a more comprehensive validation, the HT-conjugation platform needs to be further put to the test with other strains of the same or different bacterial species, other types of conjugative plasmids, and other experimental conditions, a requirement which will be fulfilled as it is being used in other conjugation studies.

## Data availability statement

The raw data supporting the conclusions of this article will be made available by the authors, without undue reservation.

## Author contributions

KA-C: Conceptualization, Data curation, Formal Analysis, Investigation, Methodology, Validation, Visualization, Writing – original draft, Writing – review & editing. AR-S: Data curation, Formal Analysis, Investigation, Methodology, Validation, Visualization, Writing – review & editing. NS-F: Methodology, Validation, Writing – review & editing. OA: Methodology, Validation, Writing – review & editing. CG: Conceptualization, Investigation, Supervision, Validation, Writing – original draft, Writing – review & editing. LA: Conceptualization, Formal Analysis, Investigation, Supervision, Writing – original draft, Writing – review & editing. IA: Conceptualization, Data curation, Formal Analysis, Funding acquisition, Investigation, Methodology, Project administration, Resources, Supervision, Writing – original draft, Writing – review & editing.
